# High-Performance Tri-Band Metamaterial Absorber for Polarization-Insensitive EMI Shielding in Microwave Communication Systems

**DOI:** 10.3390/ma19153164

**Published:** 2026-07-23

**Authors:** Iftikhar ud Din, Daud Khan, Tayeb A. Denidni

**Affiliations:** National Institute for Scientific Research (INRS), University du Quebec, Montreal, QC H5A 1K6, Canada; daud.khan@inrs.ca (D.K.); tayeb.denidni@inrs.ca (T.A.D.)

**Keywords:** metamaterial absorber, tri-band, EMI shielding, polarization-insensitive, angular stability, S-band, C-band, X-band, microwave absorption

## Abstract

A low-profile tri-band metamaterial absorber is developed for microwave attenuation and electromagnetic shielding applications within the S-, C-, and X-band regions. The absorber employs a compact resonant topology comprising a square metallic ring and two nested decagonal resonators, fabricated on an FR-4 dielectric layer with a metallic backing. Numerical optimization results in three highly efficient absorption bands located at 3.6 GHz, 7.4 GHz, and 11 GHz, where the absorptivity exceeds 99%. The physical origin of the absorption response is examined through field localization, induced current distributions, constitutive parameter extraction, and impedance characteristics. The analysis demonstrates that the resonant modes generated by the coupled metallic elements promote strong confinement of electromagnetic energy within the structure, leading to dissipation of the incident power. The geometrical unit-cell symmetry further enables a nearly identical response for different polarization states, while maintaining stable operation for incoming angles up to 60° under both TE and TM excitations. To verify the simulation results, an array prototype was manufactured and tested using a free-space characterization technique. The measured absorption characteristics closely follow the simulated response, showing the effectiveness of the design methodology. Owing to its compact dimensions, near-unity absorption, angular stability, and strong shielding capability, the developed absorber offers significant potential for electromagnetic compatibility enhancement, microwave shielding, and radar-related applications.

## 1. Introduction

The progressive expansion of modern wireless networks, satellite systems, radar platforms, Internet of Things (IoT) devices, autonomous sensing technologies, and high-frequency electronic equipment has significantly increased electromagnetic interference (EMI) across the microwave spectrum. Unwanted electromagnetic radiation can degrade communication quality, reduce system reliability, interfere with sensitive electronic circuits, and adversely affect the operation of medical, industrial, and defense-related systems. Therefore, the design of high-performance electromagnetic absorbers for mitigating undesired reflections and EMI has become increasingly important in both civilian and military sectors. Microwave absorbers play a critical role in radar cross-section (RCS) reduction, electromagnetic compatibility (EMC), stealth technology, wireless communications, sensing systems, energy harvesting, and electromagnetic shielding applications [1,2,3]. Traditional absorber technologies, such as Salisbury screens and Jaumann absorbers, generally require relatively large thicknesses and multiple dielectric layers to achieve effective absorption, thereby limiting their use in compact, lightweight platforms [4,5]. To overcome these limitations, metamaterial absorbers (MMAs) have emerged as an attractive alternative owing to their subwavelength dimensions, low-profile structures, lightweight configurations, and exceptional capability to manipulate electromagnetic waves. The theoretical foundation of metamaterials was established by Veselago’s concept of simultaneously negative permittivity and permeability [6], and subsequent developments enabled the realization of artificial electromagnetic materials with tailored constitutive parameters [7]. A major milestone was achieved by Landy et al. [8], who demonstrated the first perfect metamaterial absorber based on impedance matching between free space and a resonant structure. Since then, extensive research has been devoted to developing compact absorbers capable of achieving near-unity absorption through engineered electric and magnetic resonances that minimize reflection and maximize electromagnetic energy dissipation [9,10]. With the increasing demand for multifunctional communication, sensing, and shielding systems, significant attention has been directed toward the development of multiband metamaterial absorbers capable of supporting multiple operating frequencies within a single compact structure. Various resonator topologies, including split-ring resonators, concentric rings, complementary structures, fractal geometries, Jerusalem-cross resonators, and hybrid resonating elements, have been explored to realize multiple absorption bands with enhanced electromagnetic performance [11,12,13,14]. Recent studies have primarily focused on improving absorption efficiency, compactness, polarization stability, and angular robustness. Moniruzzaman et al. [15] reported a polarization-robust dual-band absorber for GSM and 5G EMI shielding applications, achieving absorption levels of 98.7% and 99.7% at 1.8 GHz and 3.5 GHz, respectively, while maintaining stable performance under incidence angles up to 60°. Zhu et al. [16] developed a triband absorber based on a three-ring coupled resonator configuration that generated absorption peaks of 99.72%, 99.88%, and 99.87% at 3.97 GHz, 6.85 GHz, and 10.11 GHz, respectively. Their work demonstrated that electromagnetic coupling among nested resonators can effectively generate multiple resonant modes while maintaining a compact footprint. Khalil et al. [17] introduced a multi-spectrum absorber employing combined quadrilateral and six-sided elements resonators that produced four absorption bands at 3.36 GHz, 3.64 GHz, 5.06 GHz, and 6.36 GHz. Although high absorption levels were achieved, the operating frequencies were concentrated within the lower microwave region and did not simultaneously cover the S-, C-, and X-band frequencies required by many modern communication and radar systems. More recently, Sakib et al. [18] reported a quad-band LC resonator structure-based metamaterial absorber operating at 4.685 GHz, 6.434 GHz, 8.0869 GHz, and 10.974 GHz. In addition to exhibiting strong absorption characteristics, the design was primarily developed for dielectric permittivity sensing applications using effective-medium parameter extraction and equivalent-circuit modeling. Similarly, Elalaouy et al. [19] reported an ultra-thin multi-resonance absorber operating in the 20–40 GHz millimeter-wave spectrum with absorptivities approaching 99.8%. However, the proposed structure was specifically intended for millimeter-wave applications and does not address conventional microwave-frequency EMI shielding requirements. These studies clearly demonstrate the potential of metamaterial absorbers for multiband operation; nevertheless, challenges associated with fabrication simplicity, frequency scalability, polarization stability, and practical EMI shielding performance remain significant. Despite the considerable progress achieved in recent years, several issues remain unresolved. Many reported absorbers rely on multilayer configurations, complex resonator geometries, expensive dielectric materials, or relatively large unit-cell dimensions that increase fabrication complexity and manufacturing cost. Furthermore, some structures exhibit limited angular stability, polarization dependence, closely spaced resonances, or are optimized for specialized applications such as sensing rather than electromagnetic shielding. Consequently, achieving near-unity absorption, polarization insensitivity, wide-angle stability, compact dimensions, and effective EMI shielding performance within a simple single-layer structure remains a challenging task. Absorbers capable of simultaneously covering the S-, C-, and X-band frequency regions are highly desirable because these bands are widely used in radar surveillance, space networks, wireless networks, navigation systems, and sensing technologies. Motivated by these challenges, this study proposes a compact tri-resonant absorber based on a modified nested three-resonating-element topology, implemented on an FR-4 dielectric layer. The structure function at 3.6 GHz, 7.4 GHz, and 11 GHz, corresponding to the S-, C-, and X-band frequency regions, respectively. Through careful optimization of the resonator geometry, dominant electric and magnetic responses are generated, resulting in absorption levels exceeding 99% at all operating frequencies. Owing to its geometrically symmetric configuration, the proposed structure has polarization-independent characteristics and offers a robust absorption response for both TE and TM modes when illuminated at oblique angles up to 60°. The absorption mechanism is systematically investigated through impedance matching analysis, effective medium parameter extraction, electric- and magnetic-field responses, along with induced surface-current patterns. In addition, the EMI shielding effectiveness of the presented absorber is evaluated and compared with recently reported designs to demonstrate its suitability for practical microwave communication, radar, and electromagnetic protection systems. The main achievements of this paper are outlined below:

- A compact single-layer tri-band metamaterial absorber operating at 3.6 GHz, 7.4 GHz, and 11 GHz is proposed.
- Strong absorption performance exceeding 99% is obtained at all three resonance frequencies through optimized impedance matching.
- The absorber retains robust operation under various polarization angles and stable absorption characteristics at non-normal incoming angles up to 60° for both TE and TM polarizations.
- The absorption behavior is comprehensively explained using impedance matching analysis, effective medium theory, electromagnetic field distributions, and surface current characteristics.
- Excellent EMI shielding effectiveness is achieved across the targeted frequency bands, demonstrating the suitability of the proposed design for wireless communication, radar, sensing, and electromagnetic compatibility applications.
- Unlike existing multiband absorbers, the proposed work introduces a systematic electromagnetic resonance-synthesis methodology in which each resonator is deliberately designed to generate an independent absorption mode while preserving the previously established resonances. This deterministic design approach establishes a direct relationship between the resonator topology, electromagnetic coupling, and the resulting tri-band absorption mechanism, providing physical insight beyond conventional performance-oriented studies.

The rest of this manuscript is structured as follows. Section 2 details the configuration of the designed tri-band metamaterial absorber, including the unit-cell geometry, simulation environment, and design optimization process. Section 3 presents the absorption characteristics together with the polarization-independent and angular stability performances of the absorber. Section 4 investigates the underlying absorption mechanism through electric- and magnetic-field distributions, surface-current distributions, effective electromagnetic parameter retrieval, and impedance-matching analysis. Section 5 discusses the shielding effectiveness, fabrication procedure, experimental validation, and comparative assessment with recently reported absorbers. Finally, Section 6 summarizes the major findings and concludes the paper.

## 2. Electromagnetic Analysis and Design Optimization of the Absorber Structure

**Figure 1:**
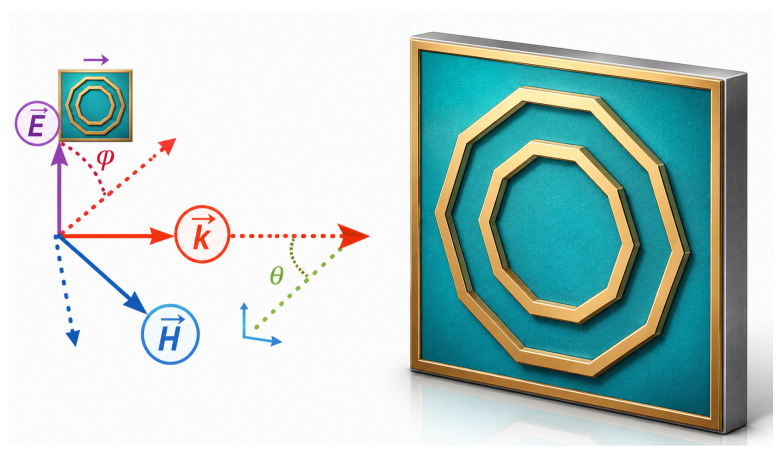
The incident electromagnetic wave and the orientations of the electric field (*E*), magnetic field (*H*), and propagation vector (*k*) with respect to the proposed absorber.

Figure 1 presents the physical structure and dimensions of the metamaterial absorber, along with the electromagnetic excitation configuration. The absorber is designed on an FR-4 dielectric layer and backed by a metallic ground layer, forming a compact structure able to obtain near-perfect absorption at three microwave frequencies. The unit cell consists of an outer square metallic ring enclosing two concentric decagonal resonators. The electromagnetic coupling between these resonant elements produces triple resonance modes, enabling efficient absorption at three distinct frequency bands. The absorption characteristics exhibit resonances at 3.6 GHz, 7.4 GHz, and 11 GHz, where the absorptivity exceeds 99% at each operating band. The excellent absorption performance is attributed to strong impedance matching with the surrounding medium, which minimizes reflected power, while the metallic ground plane suppresses transmission. Consequently, the incoming electromagnetic power is almost entirely absorbed by the design. As depicted in Figure 1, the topology is energized by an incoming plane wave characterized by the electromagnetic excitation consisting of mutually orthogonal electric and magnetic fields, denoted by *E* and *H*, respectively, together with the propagation vector *k*. The excitation conditions are determined by the wave incidence angle (θ) and polarization orientation (ϕ), enabling investigation of absorber performance under various polarization states and oblique incidence. These parameters are essential for evaluating the angular stability and polarization-insensitive characteristics of the presented design. At the resonance frequencies, enhanced field localization occurs around the metallic resonators and within the lossy dielectric substrate. This field confinement enhances dielectric and ohmic losses, thereby converting the incident power into heat. Owing to its compact geometry, simple resonator configuration, and excellent absorption performance, the presented absorber is a practical solution for microwave systems shielding, stealth applications, and electromagnetic compatibility enhancement.

## 3. Unit-Cell Design Procedure

**Figure 2:**
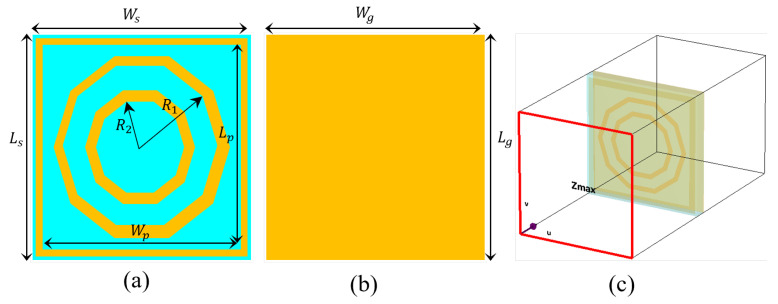
Setup of the designed MMA: (**a**) front view, (**b**) ground-plane view, and (**c**) electromagnetic wave excitation configuration.(optimized design parameters are $W_{s}=10$ mm, $L_{s}=10$ mm, $W_{p}=9.6$ mm, $L_{p}=9.6$ mm, $W_{g}=10$ mm, $L_{g}=10$ mm, $R_{1}=4$ mm, and $R_{2}=2.8$ mm).

The layout of the designed absorber is illustrated in Figure 2, where Figure 2a illustrates the front-view resonator geometry and Figure 2b depicts the metallic ground plane. The absorber is constructed with a three-layer arrangement comprising a patterned copper resonator, an FR-4 dielectric layer, and a copper backplane. The bottom surface blocks electromagnetic transmission through the structure, thereby enhancing the absorption performance. The resonating pattern is etched on the top surface, incorporating copper with an electrical conductivity of $5.8\times 10^{7}\;S/m$, loss tangent of 0.02, and a thickness of 0.035 mm. The metallic pattern supports resonant current distributions that enable efficient confinement of electromagnetic energy at the operating frequencies. A dielectric substrate composed of FR-4 material with a relative permittivity of 4.4 and a thickness of 1 mm is employed as the dielectric layer. This substrate maintains the spacing between the resonator and the bottom plane while aiding free-space impedance matching. The unit cell dimensions were optimized via parametric analysis to attain the specified tri-band absorption. The final values of the design dimensions of the optimized absorber are depicted in Figure 2.

Figure 2c illustrates the simulation configuration used to examine the electromagnetic performance of the presented tri-band metamaterial absorber. The numerical investigations were conducted in CST Microwave Studio using a frequency-domain electromagnetic analysis approach. To emulate an infinitely periodic metasurface, periodic boundary settings were imposed along the transverse (*x*–*y*) directions, while open-space boundary conditions were assigned along the propagation direction (*z*-axis). The unit cell was analyzed under the excitation of a normally incident plane wave, and its response was investigated across the frequency range of 2–12 GHz. Floquet ports were utilized to stimulate the periodic structure and extract the corresponding scattering parameters. The absorptivity of the proposed absorber is determined from the simulated scattering parameters using

(1) $$A=1-|S_{11}{|}^{2}-{\left|S_{21}\right|}^{2},$$

where $|S_{11}{|}^{2}$ and $|S_{21}{|}^{2}$ represent the reflected and transmitted power ratios, respectively. Since the design integrates a continuous metallic ground plane beneath the dielectric substrate, transmission through the absorber is effectively suppressed, resulting in $|S_{21}|\approx 0$. Consequently, the absorption can be approximated as

(2) $$A\approx 1-|S_{11}{|}^{2}.$$

This observation demonstrates that the absorption response is primarily governed by the reflection characteristics. Therefore, minimizing the reflected power through proper impedance matching enables near-unity absorption at the desired resonance frequencies, effectively suppressed, resulting in efficient absorption. The calculated absorption spectrum reveals three prominent resonance frequencies located at 3.6 GHz, 7.4 GHz, and 11 GHz. At each resonance, the absorptivity exceeds 99%, indicating excellent electromagnetic energy confinement and dissipation. The observed absorption bands arise from the combined resonant behavior of the outer square ring and the two concentric decagonal resonators, which generate distinct electromagnetic modes at different frequencies. The analysis confirms the capability of the presented metasurface to realize highly efficient tri-band absorption across the microwave frequency range.

## 4. Optimization Steps of the Presented Absorber

**Figure 3:**
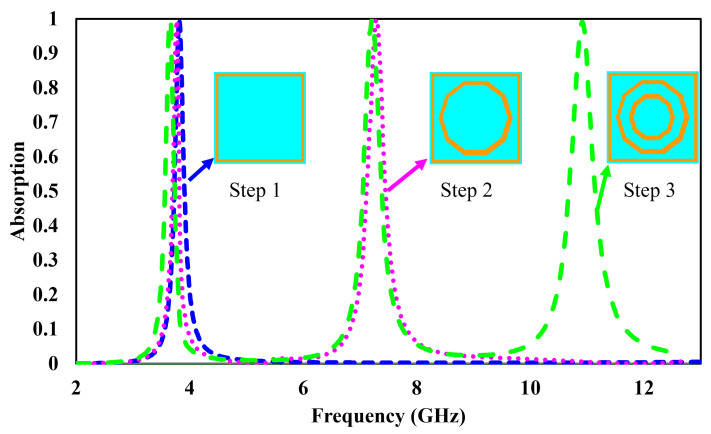
Geometric progression of absorber optimization stages and their absorption characteristics. The sequential addition of decagonal resonators progressively introduces resonant modes, resulting in the final tri-band response at 3.6 GHz, 7.4 GHz, and 11 GHz.

Figure 3 illustrates the evolution of the proposed absorber geometry and its corresponding absorption response. In Step 1, the outer square resonator generates a single absorption band centered at approximately 3.8 GHz with near-unity absorptivity. The addition of the first decagonal ring in Step 2 introduces a second resonance around 7.3 GHz while maintaining the lower-frequency absorption characteristic. In Step 3, the incorporation of a second concentric decagonal ring excites an additional resonant mode near 11 GHz, resulting in a tri-band absorption response. The progressive development demonstrates that each resonating element contributes a distinct resonance, enabling controlled generation of tri-band absorption through electromagnetic coupling. The final optimized configuration exhibits resonances at 3.6 GHz, 7.4 GHz, and 11 GHz, with absorptivity exceeding 99% at all operating frequencies. These results validate the feasibility of the proposed design method in achieving compact, high-efficiency tri-band absorption using a simple resonator topology. The 90% absorption bandwidths of the proposed absorber are 0.24 GHz (3.57–3.81 GHz), 0.34 GHz (7.28–7.62 GHz), and 0.42 GHz (10.90–11.32 GHz) for the resonances at 3.67 GHz, 7.44 GHz, and 11.00 GHz, respectively, corresponding to fractional bandwidths (FBWs) of 6.54%, 4.57%, and 3.82%. The FBW is calculated as

$$\mathrm{FBW}=\frac{f_{H}-f_{L}}{f_{c}}\times 100\%,$$

where $f_{H}$, $f_{L}$, and $f_{c}$ denote the upper, lower, and center frequencies of the corresponding absorption band, respectively.

## 5. Absorption Mechanism

**Figure 4:**
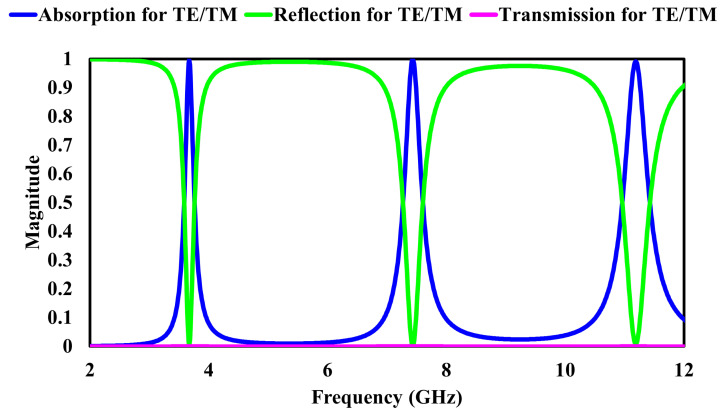
Absorption characteristics under normal incidence for transverse electric (TE) and transverse magnetic (TM) polarized waves.

Figure 4 presents the simulated reflection coefficient ($S_{11}$) and corresponding absorption response of the proposed tri-band metamaterial absorber under normal plane-wave incidence. Three distinct absorption resonances are observed at 3.6 GHz, 7.4 GHz, and 11 GHz, with peak absorptivities exceeding 99% at each operating frequency. Since the absorber is backed by a continuous metallic ground plane, the transmission coefficient is approximately zero ($S_{21}\approx 0$), and the absorption is governed primarily by the reflected power. Consequently, the strong suppression of $S_{11}$ at the three resonances results in near-unity absorption. The excellent absorption performance is attributed to effective impedance matching between the absorber and free space at the resonant frequencies, which minimizes reflection and maximizes electromagnetic energy dissipation within the lossy dielectric substrate and metallic resonators. These results confirm that the proposed resonator topology efficiently supports three independent resonant modes while maintaining compact dimensions and high absorption efficiency across the S-, C-, and X-band frequency ranges.

## 6. Polarization-Independent Absorption Response

**Figure 5:**
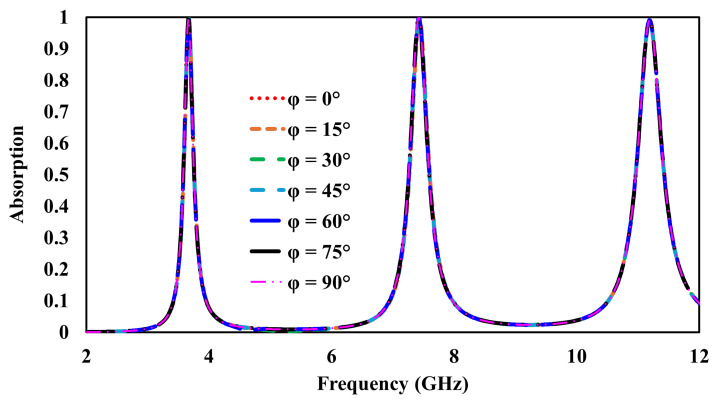
Polarization stability analysis of the presented tri-band metamaterial absorber under normal plane-wave incidence.

Figure 5 illustrates the polarization stability of the proposed tri-band metamaterial absorber under normal plane-wave incidence for polarization angles ranging from 0° to 90°. The absorption responses corresponding to different polarization angles almost completely overlap, indicating negligible variations in both the resonance frequencies and absorption magnitudes. Three distinct absorption peaks are consistently observed at 3.6 GHz, 7.4 GHz, and 11 GHz, with absorptivities exceeding 99% for all investigated polarization angles. This polarization-independent behavior is attributed to the rotational symmetry of the unit-cell geometry, which produces an identical electromagnetic response regardless of the orientation of the incident electric field. These results confirm that the proposed absorber preserves its tri-band absorption characteristics over the entire polarization range, demonstrating excellent polarization stability for practical microwave absorption and electromagnetic shielding applications.

## 7. Angular Dependent Absorption Characteristics

**Figure 6:**
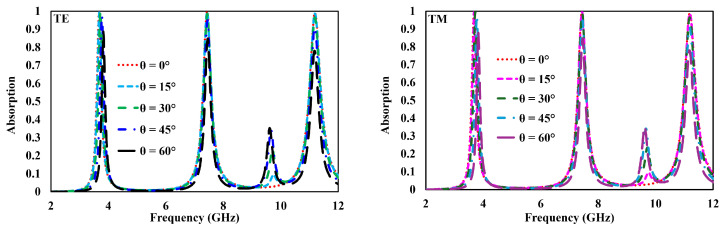
Absorptivity of the metamaterial absorber under various incidence angles (θ).

For practical applications, an absorber must maintain stable performance under oblique electromagnetic wave incidence. Therefore, the angular response of the proposed tri-band metamaterial absorber was investigated for both transverse electric (TE) and transverse magnetic (TM) polarizations at incidence angles of θ=0°, 15°, 30°, 45°, and 60°, as shown in Figure 6. The three resonant bands centered at 3.6 GHz, 7.4 GHz, and 11 GHz remain clearly distinguishable over the entire angular range. Table 1 quantitatively summarizes the resonance frequencies, peak absorptivities, and 90% absorption bandwidths for each resonant band. The results show only negligible resonance shifts together with a gradual reduction in peak absorptivity and 90% absorption bandwidth as the incidence angle increases. Nevertheless, the absorptivity remains above 80% for all three resonant bands up to 60° under both TE and TM polarizations, confirming the excellent angular stability of the proposed absorber. The slight degradation observed at larger incidence angles is attributed to changes in the incident wave vector and the tangential electric- and magnetic-field components, which slightly modify the equivalent inductive and capacitive coupling of the resonators, resulting in weaker impedance matching with free space. Despite these effects, the proposed absorber preserves its triband absorption characteristics over a wide range of incidence angles, making it well suited for practical microwave absorption and electromagnetic interference (EMI) shielding applications.

## 8. Analysis of Surface Current and Electric Field

**Figure 7:**
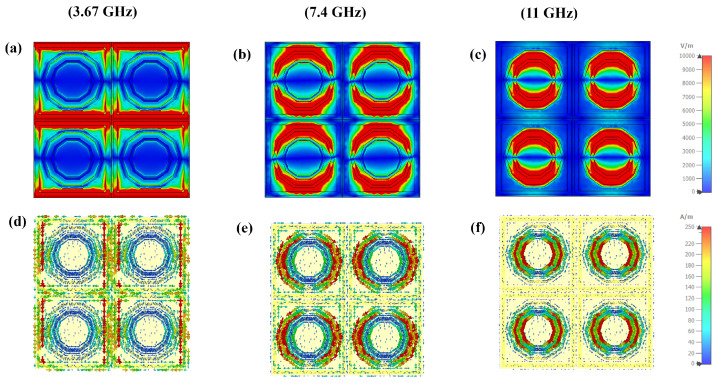
(**a**–**c**) Simulated surface electric-field intensity maps of the proposed triband absorber analyzed at the resonant frequencies of 3.67, 7.4, and 11 GHz. (**d**–**f**) Associated surface current density distributions corresponding to each absorption resonance.

Figure 7 presents the electric-field and surface-current distributions of the designed tri-band metamaterial absorber at the three resonance frequencies of 3.6 GHz, 7.4 GHz, and 11 GHz. The electric-field responses are depicted in Figure 7a–c, whereas the corresponding surface-current distributions are shown in Figure 7d–f. At 3.6 GHz, the electric field is strongly localized along the outer square resonator, notably around the corners and edge regions, as depicted in Figure 7a. This behavior shows that the lowest-frequency resonance is primarily controlled by the capacitive effect associated with the outer resonating structure. As the operating frequency shifts to a higher value of 7.4 GHz, the field intensity shifts toward the outer decagonal ring, where strong localization is observed around its perimeter, as shown in Figure 7b. At 11 GHz, the electric field is primarily confined to the inner decagonal ring, as illustrated in Figure 7c, demonstrating that this resonator generates the highest-frequency absorption mode. The surface-current distributions provide additional insight into the resonance mechanism. At 3.6 GHz, the strongest current flow is observed along the outer square resonator, as shown in Figure 7d, indicating that the longest current path contributes to the low-frequency absorption response. For the 7.4 GHz resonance, the current is primarily distributed over the outer decagonal ring, while the contribution from the square resonator becomes less significant, as depicted in Figure 7e. At 11 GHz, the current is concentrated mainly on the inner decagonal ring, where compact current loops are formed, as shown in Figure 7f. This behavior confirms the excitation of a higher-frequency resonant mode associated with a shorter current path.

## 9. The Electromagnetic Response of the Proposed Absorber Is Investigated Through the Retrieved Effective Constitutive Parameters, Including Permittivity, Permeability, and Normalized Impedance

The physical mechanism underlying the absorption performance of the designed tri-band metamaterial absorber is investigated by retrieving its effective electromagnetic parameters, namely the relative permittivity ($\varepsilon_{r}$), relative permeability ($\mu_{r}$), and normalized impedance (*Z*). These parameters provide valuable information regarding the response of the resonant structure to the incident electromagnetic wave, thereby explaining the origin of the observed absorption resonances. The effective material properties are extracted from the simulated scattering parameters using the Nicolson–Ross–Weir (NRW) retrieval approach. By employing the reflection coefficient ($S_{11}$) and transmission coefficient ($S_{21}$), the equivalent constitutive values of the absorber can be obtained. The effective permittivity and permeability are calculated as

(3) $$\varepsilon_{r}=\frac{c}{j2\pi ft}\cdot \frac{\left(1-S_{11}\right)-S_{21}}{\left(1+S_{11}\right)+S_{21}},$$

(4) $$\mu_{r}=\frac{c}{j2\pi ft}\cdot \frac{\left(1+S_{11}\right)-S_{21}}{\left(1-S_{11}\right)+S_{21}},$$

where *c* is the velocity of light in free space, while *f* represents the operating frequency, and *t* corresponds to the thickness of the substrate. Once the effective permittivity and permeability are obtained, the normalized impedance of the structure can be evaluated using

(5) $$Z=\sqrt{\frac{\mu_{r}}{\varepsilon_{r}}}.$$

**Figure 8:**
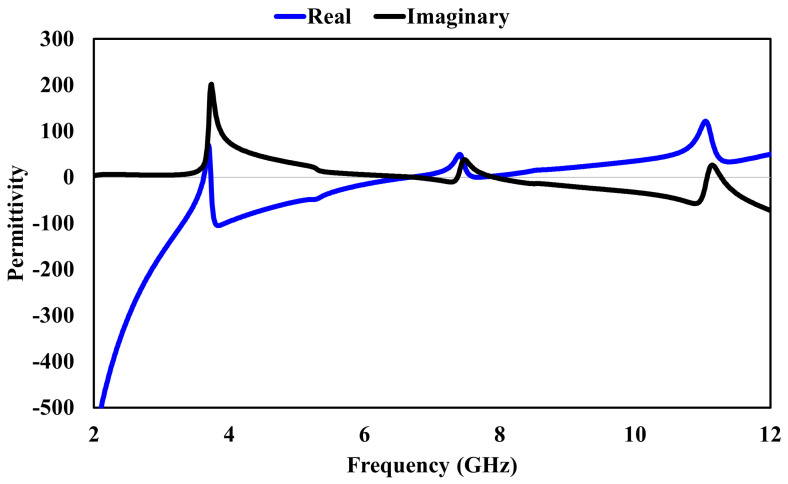
Frequency-dependent real and imaginary components of the effective permittivity for the presented absorber.

**Figure 9:**
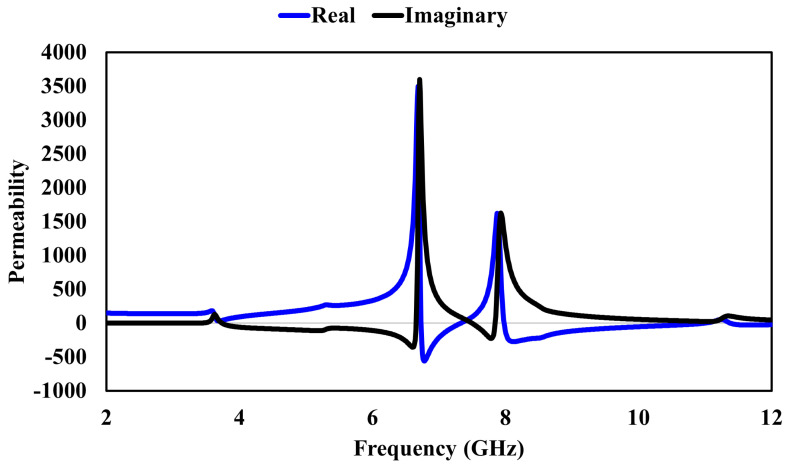
Frequency-dependent real and imaginary parts of the effective permeability for the designed absorber.

**Figure 10:**
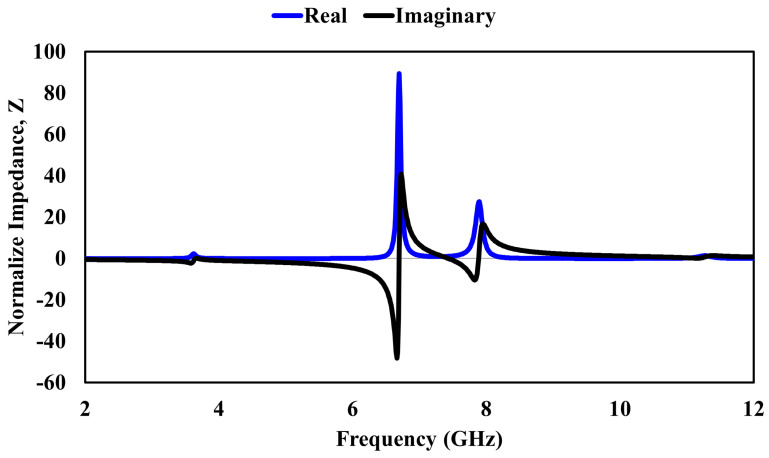
Frequency-dependent normalized impedance response of the proposed metamaterial absorber.

The retrieved constitutive parameters of the proposed metamaterial absorber are presented in Figure 8, Figure 9 and Figure 10, including the effective relative permittivity ($\varepsilon_{r}$), relative permeability ($\mu_{r}$), and normalized input impedance (*Z*). Significant dispersive behavior is observed in both $\varepsilon_{r}$ and $\mu_{r}$ in the vicinity of the absorption frequencies at 3.6, 7.4, and 11 GHz, indicating the excitation of multiple electromagnetic resonances within the structure. Moreover, the corresponding imaginary components exhibit pronounced peaks near these frequencies, reflecting increased dielectric and magnetic losses. Such losses enhance the attenuation of incident electromagnetic waves and play a vital role in the overall absorption process. To gain further insight into the absorption mechanism, the impedance-matching characteristics of the absorber are examined. High absorption can be achieved when the input impedance of the structure approaches the characteristic impedance of free space, thereby suppressing unwanted reflections. The reflection coefficient is defined as

(6) $$S_{11}=\left|\frac{Z/Z_{0}-1}{Z/Z_{0}+1}\right|,$$

where $Z_{0}=377\;\Omega$ denotes the intrinsic impedance of free space. As illustrated in Figure 10, the normalized impedance approaches unity while its imaginary part remains close to zero at 3.6, 7.4, and 11 GHz. This behavior confirms effective impedance matching between the absorber and free space, resulting in a substantial reduction in reflected energy. Consequently, the incident electromagnetic waves are efficiently coupled into the structure and dissipated through dielectric and ohmic losses, leading to near-unity absorption at all three operating frequencies.

## 10. Shielding Effectiveness and EMI Suppression Characteristics of the Proposed Tri-Band Metamaterial Absorber

**Figure 11:**
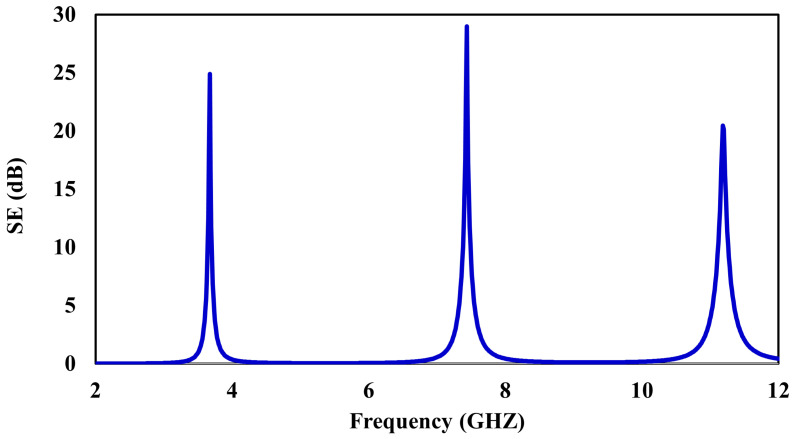
Shielding effectiveness response of the proposed tri-band metamaterial absorber, showing enhanced attenuation at 3.6 GHz, 7.4 GHz, and 11 GHz.

The continuous expansion of wireless communication infrastructures, radar systems, and high-frequency electronic technologies has intensified concerns related to electromagnetic interference (EMI). Uncontrolled electromagnetic radiation can adversely affect sensitive electronic components, degrade communication quality, and reduce the operational reliability of modern electronic systems [20]. Consequently, considerable attention has been directed toward compact electromagnetic absorbers capable of suppressing unwanted radiation while improving electromagnetic compatibility. The shielding effectiveness (SE) is defined as the attenuation of the transmitted electromagnetic field and is expressed as [21]

(7) $$SE=20\log_{10}\left(\frac{E_{I}}{E_{T}}\right),$$

where $E_{I}$ and $E_{T}$ denote the incident and transmitted electric fields, respectively. This expression quantifies the reduction of the transmitted electromagnetic wave due to the shielding structure. It should be noted that the proposed absorber incorporates a continuous metallic ground plane, which effectively blocks electromagnetic transmission and therefore provides the primary contribution to the overall shielding effectiveness. Consequently, the reported SE should not be attributed solely to the patterned resonator. Instead, the resonant metasurface enhances the absorption performance by suppressing reflection through impedance matching and converting the incident electromagnetic energy into dielectric and ohmic losses at the designed resonant frequencies. Therefore, the overall electromagnetic attenuation results from the combined action of the metallic ground plane, which suppresses transmission, and the patterned resonator, which minimizes reflection and maximizes absorption. As illustrated in Figure 11, the proposed absorber exhibits three pronounced resonant absorption bands at 3.6 GHz, 7.4 GHz, and 11 GHz, with shielding effectiveness exceeding 20 dB. These resonances correspond to efficient impedance matching and enhanced electromagnetic energy dissipation within the resonator and dielectric substrate, demonstrating that the resonant metasurface complements the metallic backplane by improving absorption rather than providing transmission shielding.

## 11. Equivalent Circuit Model (ECM) of the Proposed Absorber

**Figure 12:**
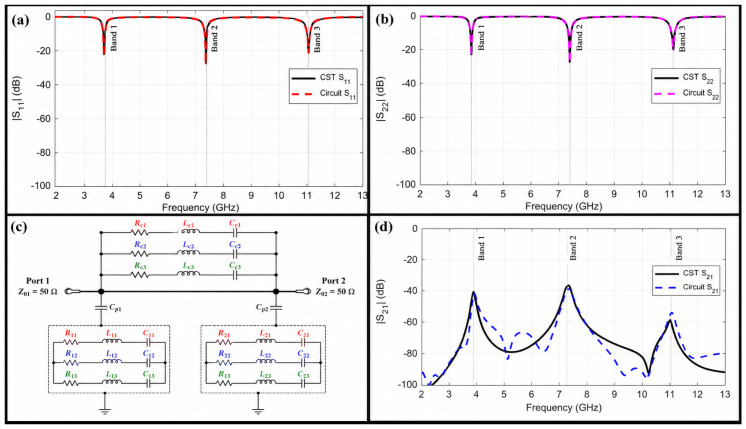
Equivalent circuit validation of the proposed triband two-port structure: (**a**) $S_{11}$, (**b**) $S_{22}$, (**c**) optimized lumped-element equivalent circuit model, and (**d**) $S_{21}$. The equivalent circuit closely matches the full-wave CST results across the three operating bands.

To provide a deeper physical understanding of the proposed triband two-port structure, an equivalent lumped-element circuit model was developed using optimized resistor–inductor–capacitor (RLC) networks, as illustrated in Figure 12. Each port is modeled by a shunt parasitic capacitance ($C_{p1}$ and $C_{p2}$) together with three parallel series-RLC branches connected to ground, representing the three resonant modes responsible for the reflection responses ($S_{11}$ and $S_{22}$). The electromagnetic coupling between the two ports is modeled by three additional series-RLC branches connected between the port nodes, which accurately reproduce the transmission response ($S_{21}$).The values of the equivalent circuit components were extracted through parameter optimization to achieve close agreement between the circuit response and the full-wave electromagnetic simulation across the entire operating frequency range. The optimized component values are summarized in Table 2. Each RLC branch corresponds to a distinct resonant mode of the proposed structure, while the coupling branches characterize the mutual electromagnetic interaction between the two ports. The excellent agreement between the equivalent circuit model and the full-wave simulation confirms the validity of the proposed operating mechanism. Furthermore, the developed circuit model provides an efficient analytical tool for rapid design optimization, parametric investigation, and physical interpretation of the triband electromagnetic response.

## 12. Measured Results

**Figure 13:**
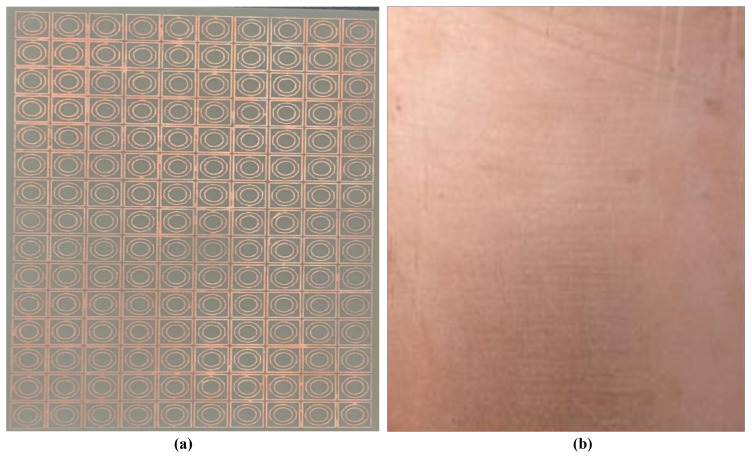
Fabrication prototype of the proposed metamaterial absorber: (**a**) front view and (**b**) back view.

**Figure 14:**
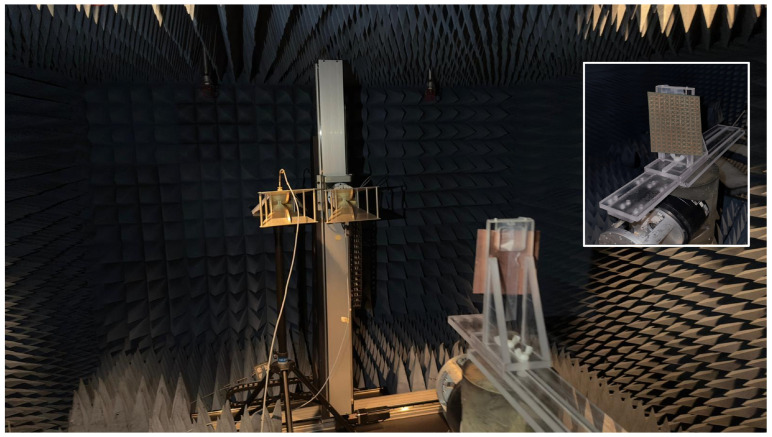
Free-space measurement setup used for the experimental evaluation of the proposed absorber.

**Figure 15:**
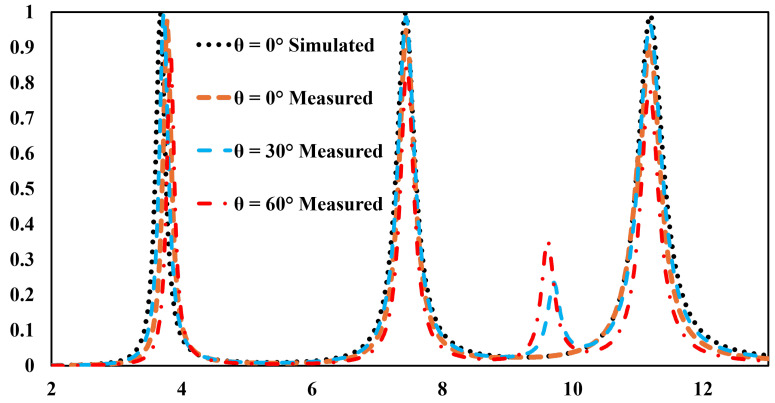
Measured absorption responses of the proposed tri-band metamaterial absorber at incidence angles of 0°, 30°, and 60°, demonstrating stable absorption characteristics under oblique incidence responses.

To experimentally validate the proposed tri-band metamaterial absorber, a prototype was fabricated and characterized using a free-space measurement setup. The fabricated sample, shown in Figure 13, consists of a 10×15 array of unit cells with an overall size of 100×150 mm^2^. This array size was selected to minimize edge effects and closely approximate the response of an infinitely periodic structure. The experimental setup is illustrated in Figure 14. A vector network analyzer (VNA) connected to two broadband horn antennas was used to measure the reflection characteristics over the frequency range of 2–12 GHz. The transmitting and receiving antennas were positioned in the far-field region to ensure uniform illumination of the sample and accurate acquisition of the reflected signals. Prior to the measurements, a metallic copper plate having the same dimensions as the fabricated absorber was employed as the reference target. The reflected response from the copper plate, corresponding to nearly perfect reflection, was used to calibrate and normalize the measured data. Following calibration, the fabricated absorber replaced the reference plate without changing the measurement geometry. Reflection measurements were first performed under normal incidence (θ=0°) and subsequently repeated for oblique incidence angles of θ=30° and θ=60° by rotating the sample while maintaining the same antenna configuration. The measured reflection coefficients were then converted into absorptivity values and compared with the simulated response, as presented in Figure 15. The experimental results exhibit good agreement with the simulated response for all three incidence angles. At normal incidence, the three absorption resonances are accurately reproduced with only minor frequency deviations attributed to fabrication tolerances, substrate parameter variations, and measurement uncertainties. As the incidence angle increases to 30° and 60°, the three resonances remain clearly observable with only a slight reduction in peak absorptivity and negligible resonance-frequency shifts, confirming the excellent angular stability of the proposed absorber. The close agreement between simulation and experiment verifies the effectiveness of the proposed design and demonstrates its robustness under both normal and oblique incidence.

## 13. Comparative Study with Previously Published Metamaterial Absorbers

Table 3 describes a comparison between the presented metamaterial absorber and recently existing polarization-insensitive absorbers in terms of unit-cell size, substrate thickness, resonance frequencies, absorption performance, and use cases. It can be seen that most reported designs achieve high absorption by employing either larger unit cells, multiple resonating elements, or operation at higher microwave frequencies. In comparison to prior studies, the presented absorber achieves a compact unit cell of only $10\times 10\;{\mathrm{mm}}^{2}$ while maintaining excellent absorption characteristics. Compared with the absorbers reported in [15,22,23,24,25,26,27,28,29], the proposed design provides three distinct absorption bands at 3.67 GHz, 7.44 GHz, and 11 GHz, with absorptivity exceeding 99.9% at all resonances. Although several existing works demonstrate multiband operation, their absorption levels are generally lower, their structures occupy larger dimensions, or they operate within limited frequency regions. The proposed absorber simultaneously covers the S-, C-, and X-band frequency ranges using a simple geometry composed of a square resonator and concentric decagonal rings. Furthermore, unlike some reported absorbers that are sensitive to polarization or require relatively large unit-cell dimensions, the proposed structure maintains polarization-insensitive behavior while preserving a compact footprint and low-profile configuration. The combination of high absorption efficiency, compact size, tri-band operation, and simple structural topology demonstrates the effectiveness of the proposed design for practical microwave absorption, electromagnetic shielding, and electromagnetic compatibility applications.

## 14. Conclusions

This work presents the design, implementation, and experimental verification of a compact tri-band metamaterial absorber intended for microwave absorption and electromagnetic interference (EMI) mitigation. The proposed configuration consists of an outer square resonator integrated with two concentric decagonal rings on an FR-4 substrate, producing three distinct absorption resonances at 3.6 GHz, 7.4 GHz, and 11 GHz. Near-unity absorption exceeding 99% is achieved at all operating bands as a result of efficient impedance matching and enhanced localization of electromagnetic energy within the resonant elements. The underlying absorption phenomena were examined through electric-field distributions, surface-current profiles, and effective electromagnetic parameter retrieval. The analysis reveals that the three resonances originate from different resonating sections of the unit cell, each contributing to a specific absorption band. The evolution of the field and current distributions confirms the excitation of distinct resonant modes responsible for the observed multi-band behavior. The proposed absorber also demonstrates robust performance under both transverse-electric (TE) and transverse-magnetic (TM) excitations. Furthermore, strong absorption is preserved for incident angles up to 60°, indicating excellent polarization independence and angular resilience. These characteristics ensure reliable operation under practical electromagnetic environments where wave polarization and arrival direction may vary. Experimental characterization carried out using a free-space measurement arrangement shows strong agreement with the simulated response, validating the design methodology and numerical analysis. Combining compact dimensions, high absorption efficiency, stable wide-angle performance, and effective EMI shielding capability, the proposed absorber offers considerable potential for electromagnetic compatibility enhancement, interference suppression, radar cross-section reduction, and microwave protection applications across the S-, C-, and X-band frequency regions. 

## Figures and Tables

**Table 1:** Quantitative angular stability of the proposed tri-band metamaterial absorber.

Incidence Angle	Resonance 1			Resonance 2			Resonance 3		
	Freq.	Peak	90% BW	Freq.	Peak	90% BW	Freq.	Peak	90% BW
	(GHz)	(%)	(GHz)	(GHz)	(%)	(GHz)	(GHz)	(%)	(GHz)
0°	3.67	99.9	0.24	7.44	99.9	0.34	11.00	99.8	0.42
15°	3.67	99.5	0.23	7.44	99.4	0.33	11.02	98.8	0.40
30°	3.66	98.6	0.22	7.43	98.5	0.31	11.03	96.5	0.37
45°	3.65	94.5	0.19	7.42	93.8	0.27	11.05	89.0	0.30
60°	3.63	86.0	0.14	7.40	85.0	0.21	11.07	80.5	0.22

**Table 2:** Optimized lumped-element values of the equivalent circuit model.

Section	Branch	Resistance (Ω)	Inductance (nH)	Capacitance (pF)
Coupling Network	1	$R_{c1}=0.767$	$L_{c1}=0.622$	$C_{c1}=0.930$
	2	$R_{c2}=0.873$	$L_{c2}=0.622$	$C_{c2}=0.377$
	3	$R_{c3}=1.792$	$L_{c3}=2.918$	$C_{c3}=0.721$
Port 1 Shunt	1	$R_{11}=0.057$	$L_{11}=0.135$	$C_{11}=43.149$
	2	$R_{12}=0.124$	$L_{12}=0.159$	$C_{12}=5.888$
	3	$R_{13}=0.507$	$L_{13}=0.235$	$C_{13}=1.207$
Port 2 Shunt	1	$R_{21}=258.539$	$L_{21}=54.222$	$C_{21}=0.0158$
	2	$R_{22}=80.936$	$L_{22}=150.000$	$C_{22}=80.000$
	3	$R_{23}=0.001$	$L_{23}=3.186$	$C_{23}=0.0048$
Port 1 Coupling Cap.	–	–	–	$C_{p1}=2.873$
Port 2 Coupling Cap.	–	–	–	$C_{p2}=6.104$
Port Impedance	Port 1	$Z_{01}=50\;\Omega$	–	–
Port Impedance	Port 2	$Z_{02}=50\;\Omega$	–	–

**Table 3:** Comparison of the proposed absorber with related polarization-insensitive absorbers.

Ref.	Year	UC Size	Sub.	Freq. (GHz)	Abs. (%)	PI	Application
[15]	2023	20×20	1.6	2.4, 3.5, 5.8	99.3, 95.6, 99.5	Yes	WLAN, 5G
[22]	2019	10×10	1.6	5.57, 7.97, 13.44	98.9, 97.9, 99.28	Yes	Radar detection
[23]	2019	24×24	1.6	8.6, 10.2, 11.95	>80	Yes	X/Ku bands
[24]	2022	28.3×28.3	1.5	2.4, 5.2, 5.8	99.98	No	Energy harvesting
[25]	2023	17×17	1.6	2.03, 8.25, 12	97.8, 99.3	Yes	5G mm-wave
[26]	2021	10.4×10.4	1.6	3.2, 5.32, 11.15, 16.73	95.7, 97.7	Yes	S/C/X bands
[27]	2022	8×8	0.8	24, 28	98.94	No	5G mm-wave
[28]	2025	24×24	1.6	1.8, 3.5	98.7, 99.7	Yes	EMI shielding
[29]	2026	10×10	1.0	5.4, 9.65, 15.73	96.9, 98, 98	Yes	C/X/Ku bands
**Prop.**	**2026**	10×10	**1.0**	**3.67, 7.44, 11**	**99.9, 99.9, 99.9**	**Yes**	**S/C/X bands**

## Data Availability

The original contributions presented in this study are included in the article. Further inquiries can be directed to the corresponding author.
